# Supplementary figures and images for: A bacterial effector protein prevents MAPK-mediated phosphorylation of SGT1 to suppress plant immunity
Source: PLoS Pathog. 2020 Sep 25;16(9):e1008933. doi: 10.1371/journal.ppat.1008933 (PMC7540872; doi:10.1371/journal.ppat.1008933)

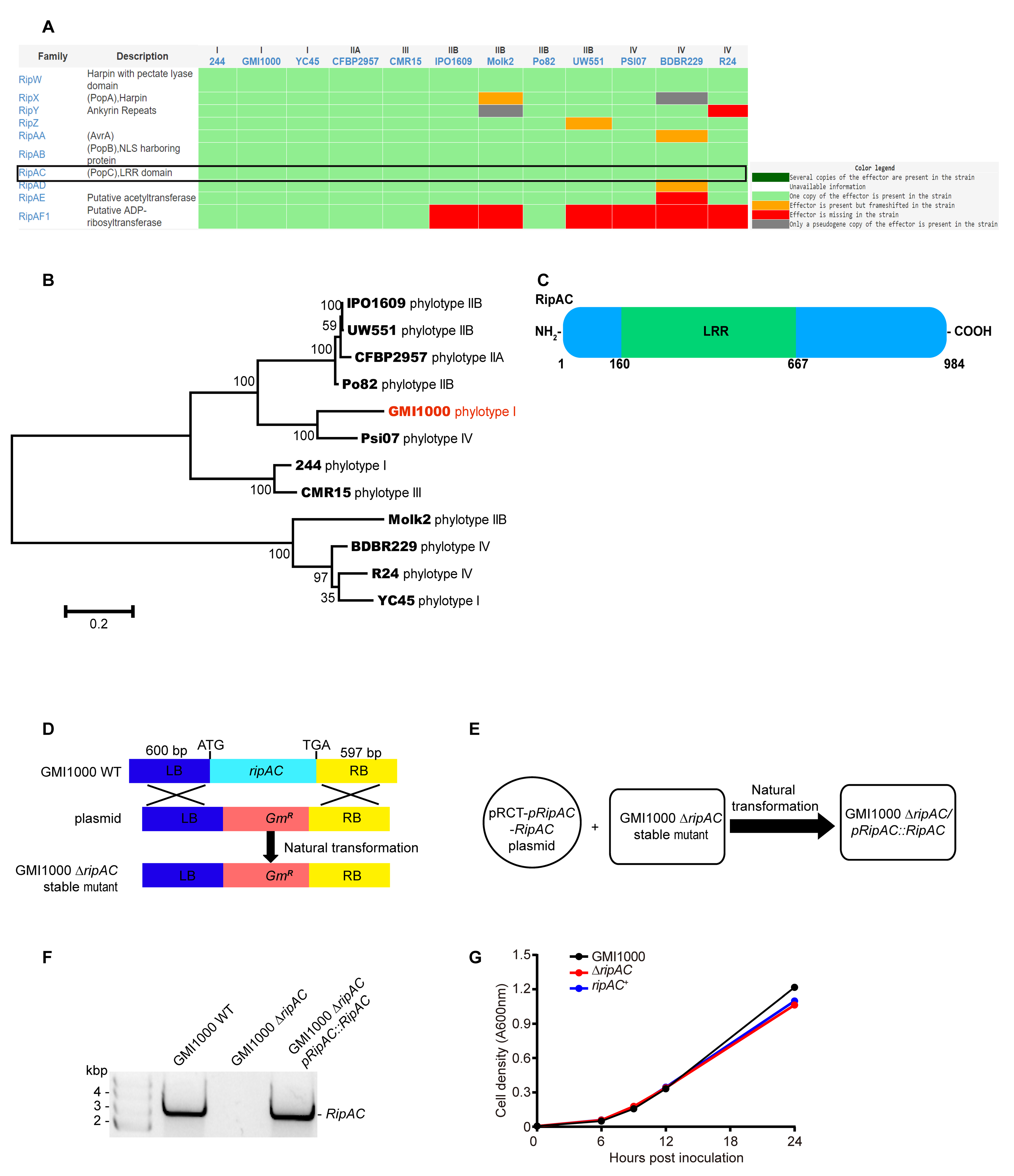

Supplement: S1 Fig — (A) RipAC is a core effector present in most sequenced R. solanacearum strains. The data is retrieved from the Ralsto T3E website (https://iant.toulouse.inra.fr/bacteria/annotation/site/prj/T3Ev3/). (B) Phylogenetic analysis of RipAC proteins from different sequenced R. solanacearum strains. The phylogenetic tree was generated using the Maximum Likelihood method based on the JTT matrix-based model. The tree is drawn to scale with branch lengths meaning the measurement number of substitutions per site. (C) RipAC encodes a leucine rich repeat protein with no predicted enzymatic domain. The upper panel is the diagram of the RipAC protein with LRR domain; the lower panel is the detailed analysis of the RipAC LRR domain, and the numbers on the left indicate LRR numbers. (D) Diagram of the process of generation of the R. solanacearum ΔripAC mutant. The ΔripAC mutant was generated by homologous recombination method using a pEASYBLUNT-based plasmid as described in the methods section. (E) Generation of RipAC complementation strain in the ΔripAC mutant strain. A 423bp DNA fragment upstream of ATG of the RipABC operon was amplified and inserted into pRCT plasmid, and the RipAC coding region was shifted into the pRCT plasmid by LR reaction to result in the pRCT-pRipAC-RipAC expression cassette. The integrative pRCT-pRipAC-RipAC plasmid was mobilized into the ΔripAC mutant by natural transformation to result in ripAC+. (F) PCR characterization of the presence of the RipAC DNA fragment in different strains. A pair of PCR primers was designed to amplify the RipAC full-length coding region and the PCR was performed to examine the presence of RipAC gene. (G) Bacterial growth in nutrient-rich medium. GMI1000 WT, ΔripAC, and ripAC+ strains were inoculated into the complete BG liquid medium with initial OD600 = 0.005 and the bacterial growth was monitored at the indicated time points measuring OD600 (mean ± SEM, n = 3). (TIF) [file ppat.1008933.s001.tif]

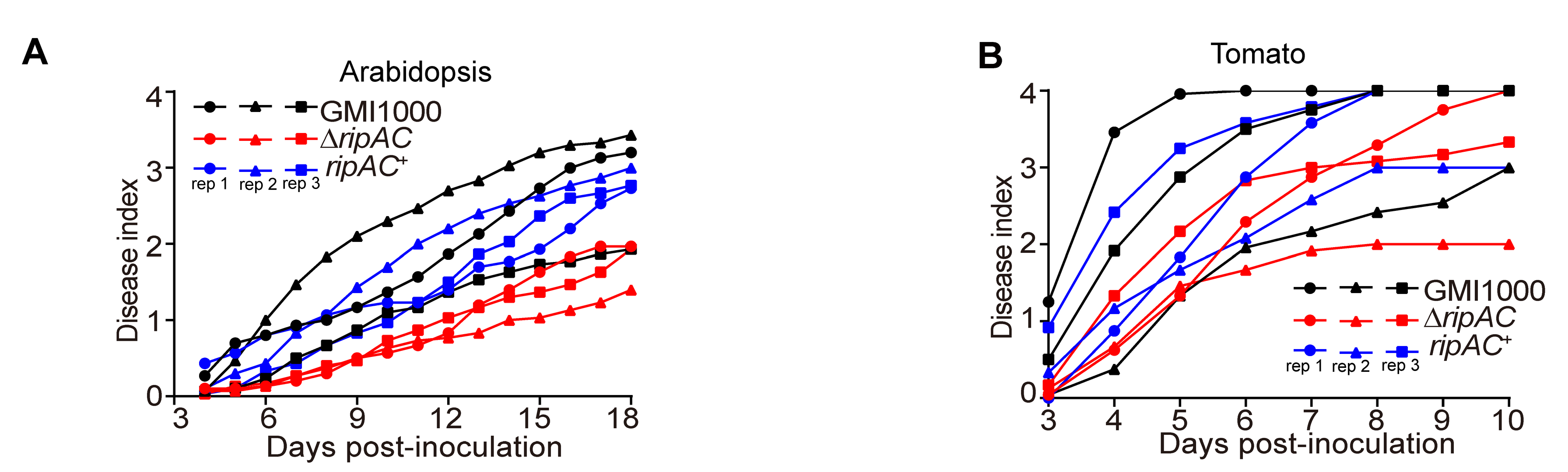

Supplement: S2 Fig — (A) Soil-drenching inoculation assays in Arabidopsis were performed with GMI1000 WT, ΔripAC mutant, and RipAC complementation (ripAC+) strains. Composite data from 3 independent biological repeats (average values are shown in Fig 1A). n = 15 plants per genotype in each repeat. (B) Soil-drenching inoculation assays in tomato were performed with GMI1000 WT, ΔripAC mutant, and RipAC complementation (ripAC+) strains. Composite data from 3 independent biological repeats (average values are shown in Fig 1C). n = 12 plants per genotype in each repeat. (TIF) [file ppat.1008933.s002.tif]

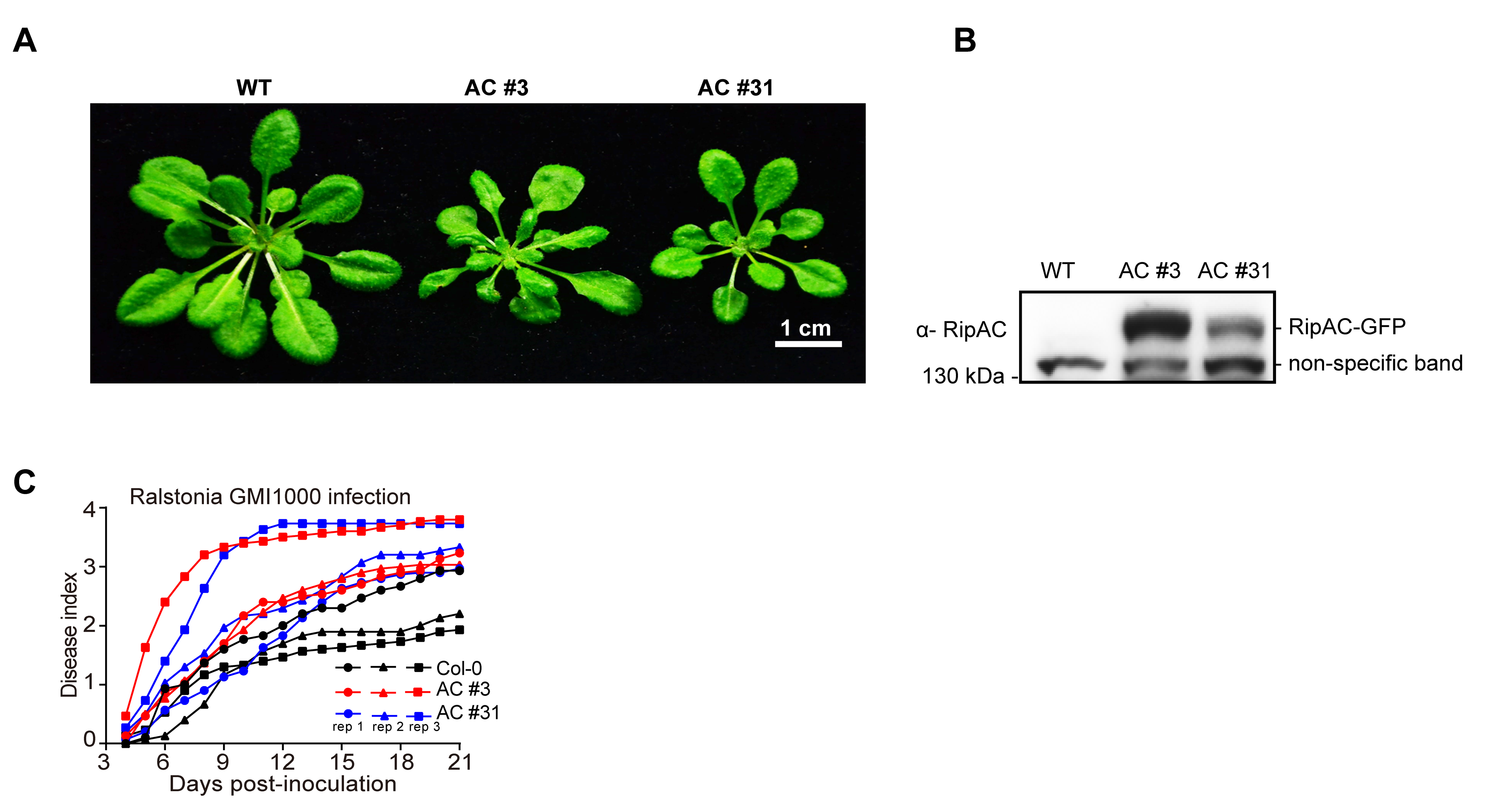

Supplement: S3 Fig — (A) Typical developmental phenotypes of RipAC-GFP transgenic Arabidopsis. AC #3 and AC #31 are two independent transgenic lines (T4 generation). The picture shows 1-month-old Arabidopsis grown in a short-day growth chamber. (B) Western blot shows RipAC-GFP protein accumulation in transgenic Arabidopsis. Samples were taken at 12 days after germination. Blots were probed with antibody Anti-RipAC (1,5000). (C) Soil-drenching inoculation assays in RipAC-GFP transgenic lines with GMI1000 WT strain. Composite data from 3 independent biological repeats (average values are shown in Fig 1A). n = 15 plants per genotype in each repeat. (TIF) [file ppat.1008933.s003.tif]

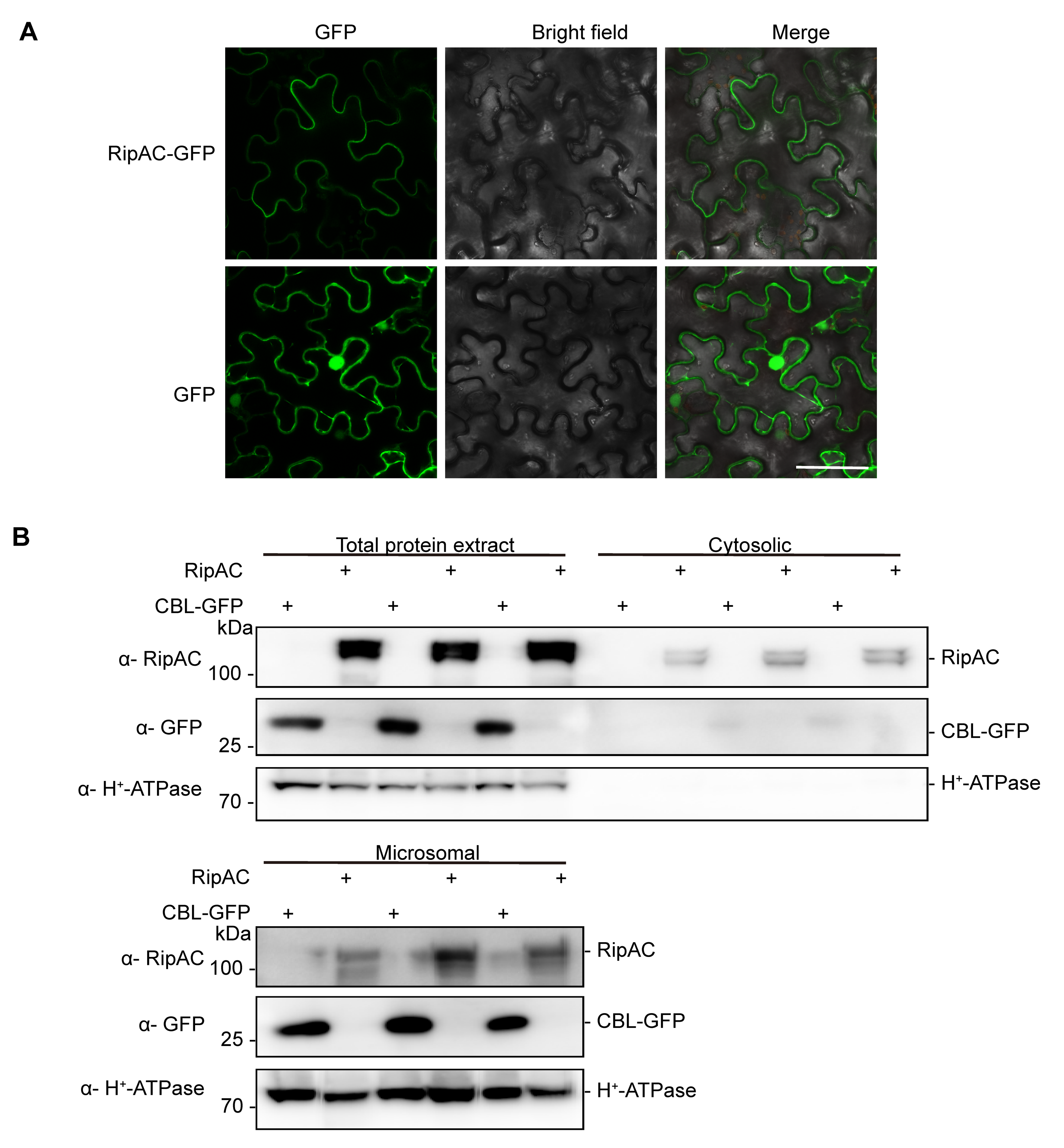

Supplement: S4 Fig — (A) Subcellular localization of RipAC-GFP in Nicotiana benthamiana. RipAC-GFP or free GFP were transiently expressed in N. benthamiana leaves using Agrobacterium tumefaciens, and the GFP fluorescence signal was observed 48 hpi using confocal microscopy. Scale bar = 50 μm. (B) Microsome fractionation in N. benthamiana. Agrobacterium carrying RipAC or CBL-GFP was infiltrated into 5-week-old N. benthamiana leaves and samples were taken at 2dpi and then subjected to microsome fractionation. The total protein extraction was separated into the cytosolic fraction and the microsomal fraction using centrifugation as described in the methods section. Protein samples from total extract, cytosolic, and microsome fraction were used for western blot. The plasma-membrane protein H+-ATPase was used as a microsomal protein marker. Western blots from 3 biological replicates are represented. (TIF) [file ppat.1008933.s004.tif]

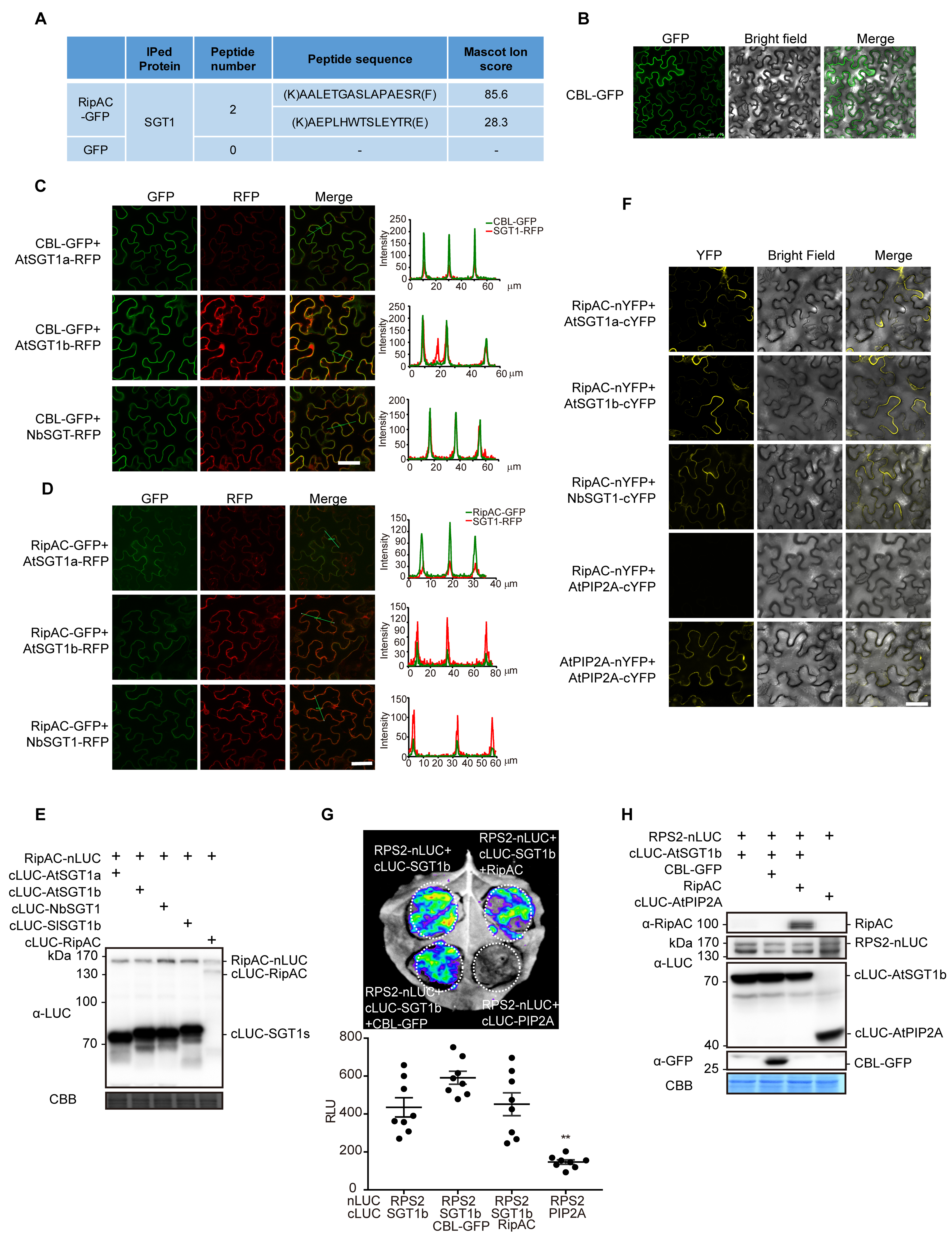

Supplement: S5 Fig — (A) RipAC-GFP or free GFP were transiently expressed in Nicotiana benthamiana leaves. The figure shows the unique NbSGT1 peptides identified exclusively in RipAC-GFP sample upon GFP immunoprecipitation followed by IP-MS/MS analysis. (B) CBL-GFP localizes at plasma membrane in N. benthamiana. bar = 75 μm. (C) Co-localization analyses of plasma membrane-associated CBL-GFP protein and SGT1-RFP in N. benthamiana. (D) RipAC-GFP co-localizes with SGT1-RFP in N. benthamiana. In (B), (C) or (D) CBL-GFP alone, CBL-GFP with SGT1-RFP (AtSGT1a, AtSGT1b, and NbSGT1) or RipAC-GFP with SGT1-RFP (AtSGT1a, AtSGT1b, and NbSGT1) was transiently expressed using Agrobacterium in N. benthamiana, and the GFP fluorescence signal was observed 48 hpi using confocal microscopy. In (C) and (D), the images show the fluorescence from GFP, RFP channel, and the merged fluorescence from both channels. The corresponding fluorescence intensity profiles (GFP, green; RFP, red) across the green lines are shown. Scale bar = 50 μm. (E) Western blot showing protein accumulation in Fig 2B. (F) Split-YFP complementation assay to determine direct interaction between RipAC and SGT1 in N. benthamiana. The self-association of aquaporin AtPIP2A was used as a positive interaction control, while the RipAC-AtPIP2A combination was used as a negative control. Fluorescence signal was observed 48 hpi using confocal microscopy. Scale bar = 50 μm. (G) Competitive Split-LUC showing that RipAC does not interfere with RPS2-SGT1b association in N. benthamiana. (H) Western blot shows the protein accumulation in (G). The RPS2-PIP2A combination was used as negative control. In all the competitive interaction assays, in addition to the interaction pair, RipAC or CBL-GFP (as negative control) were expressed to determine interference. In (G) luciferase activity was determined both qualitatively (CCD camera, higher panel) and quantitatively (microplate luminescence reader, lower panel). All the experiments were performed 3 times wit [file ppat.1008933.s005.tif]

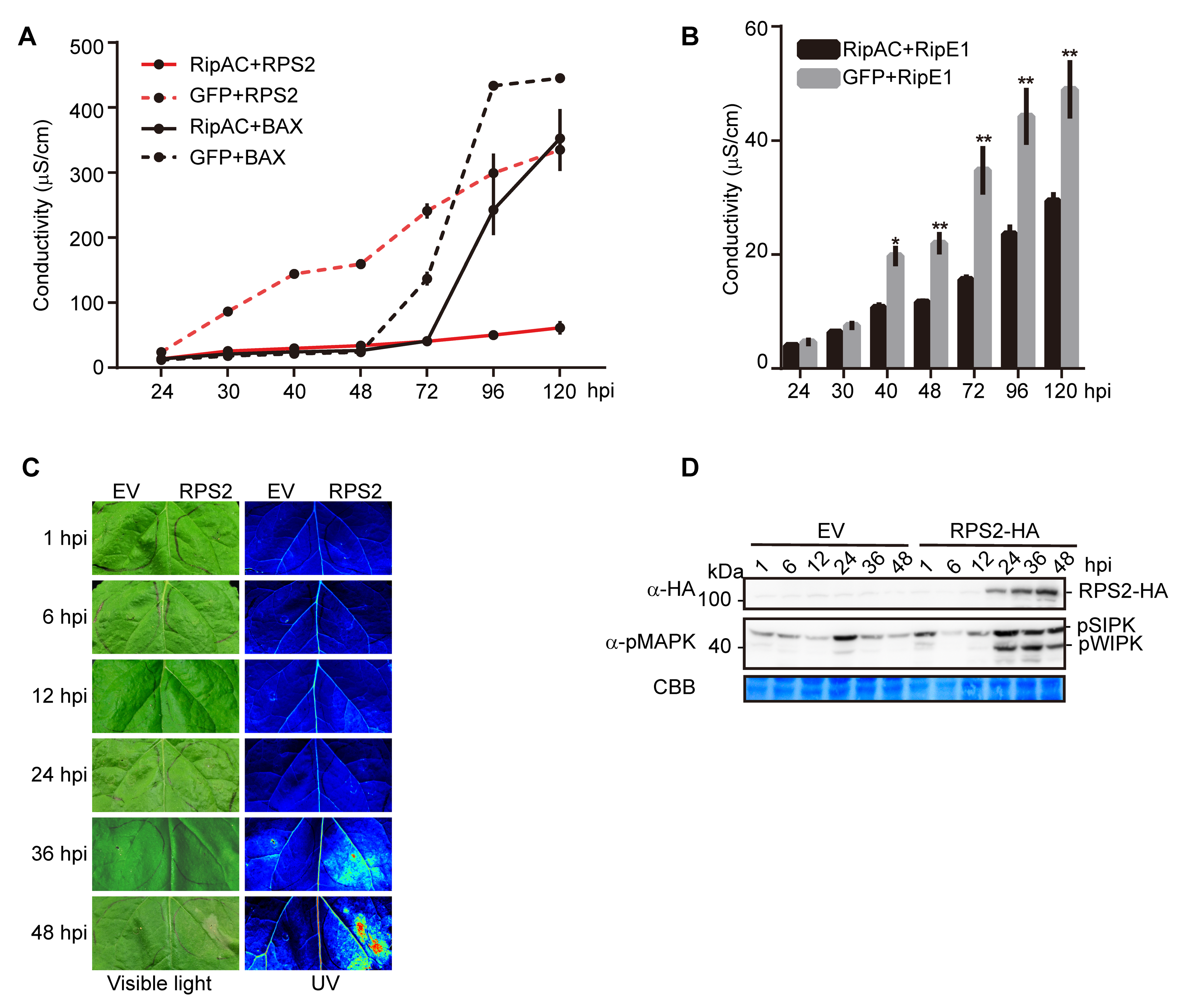

Supplement: S6 Fig — (A) Ion leakage assays showing RipAC specifically suppresses RPS2-, but not BAX-, mediated cell death in Nicotiana benthamiana. Agrobacterium expressing RipAC or the GFP control (OD600 = 0.5) were infiltrated into N. benthamiana leaves 1 day before infiltration with Agrobacterium expressing RPS2 or BAX (OD600 = 0.15). Leaf discs were taken 21 hpi for conductivity measurements at the indicated time points. The time points in the x-axis are indicated as hpi with Agrobacterium expressing RPS2 or BAX (mean ± SEM, n = 3, 4 replicates). (B) Ion leakage assays showing RipAC suppresses RipE1-mediated cell death in N. benthamiana. The ion leakage assays were performed the same as in (A) (mean ± SEM, n = 3, * p<0.05, ** p<0.01, t-test, 3 replicates). (C) RPS2-triggered cell death in N. benthamiana was monitored by visible light and UV light. (D) Phosphorylation of NbSIPK and NbWIPK was detected using anti-pMAPK antibody in (C). (TIF) [file ppat.1008933.s006.tif]

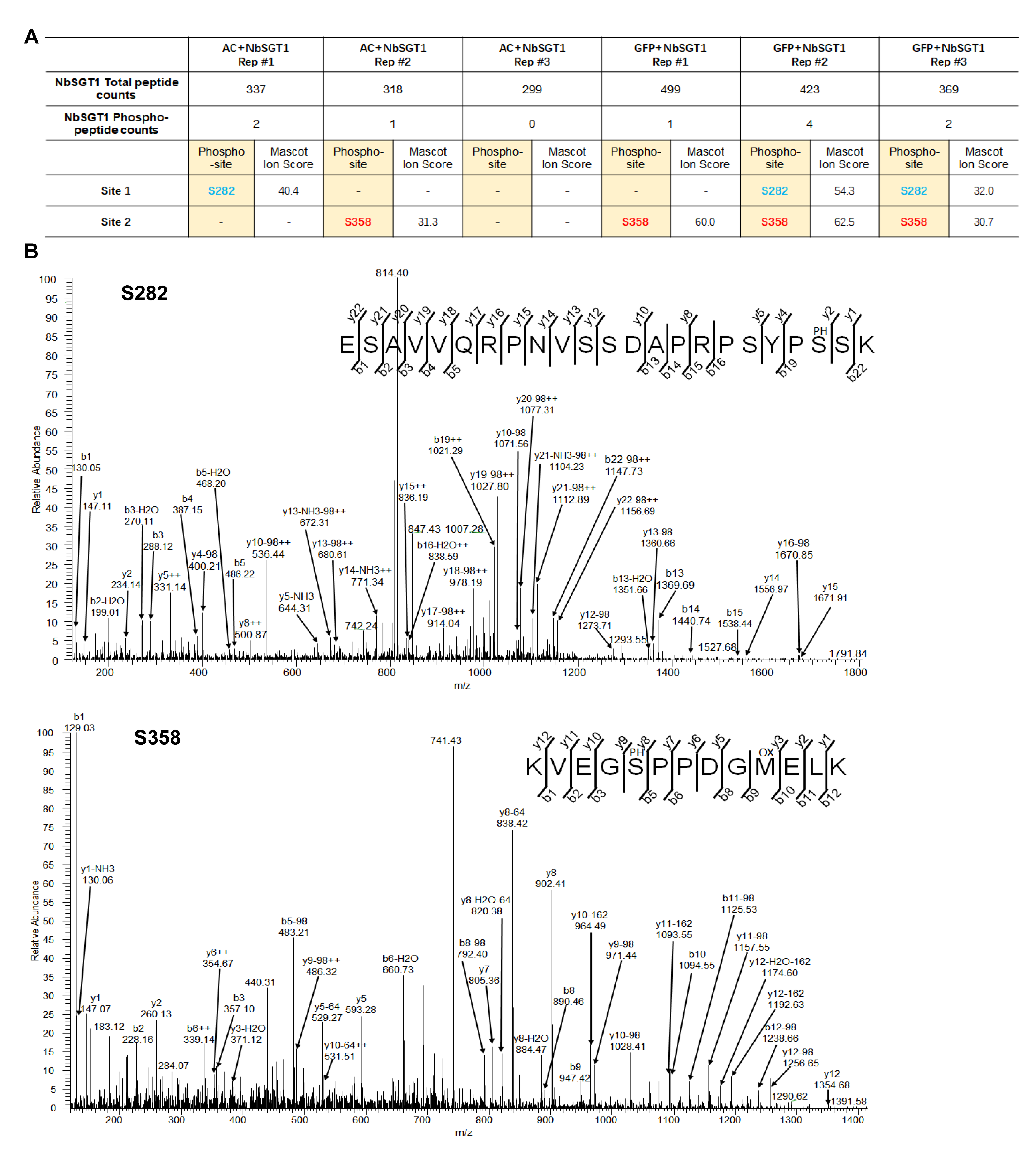

Supplement: S7 Fig — (A) NbSGT1 is phosphorylated in Nicotiana benthamiana. Agrobacterium containing NbSGT1-FLAG with RipAC or GFP was infiltrated into 4–5 weeks old N. benthamiana plants and samples were harvested 48 hpi and were subjected to anti-FLAG IP-MS/MS. The phosphorylation of S282 and S358 is summarized from three biological IP-MS/MS replicates. (B) Representative MS/MS spectra showing phosphorylation of Ser282 and Ser358 in NbSGT1 expressed in N. benthamiana. (TIF) [file ppat.1008933.s007.tif]

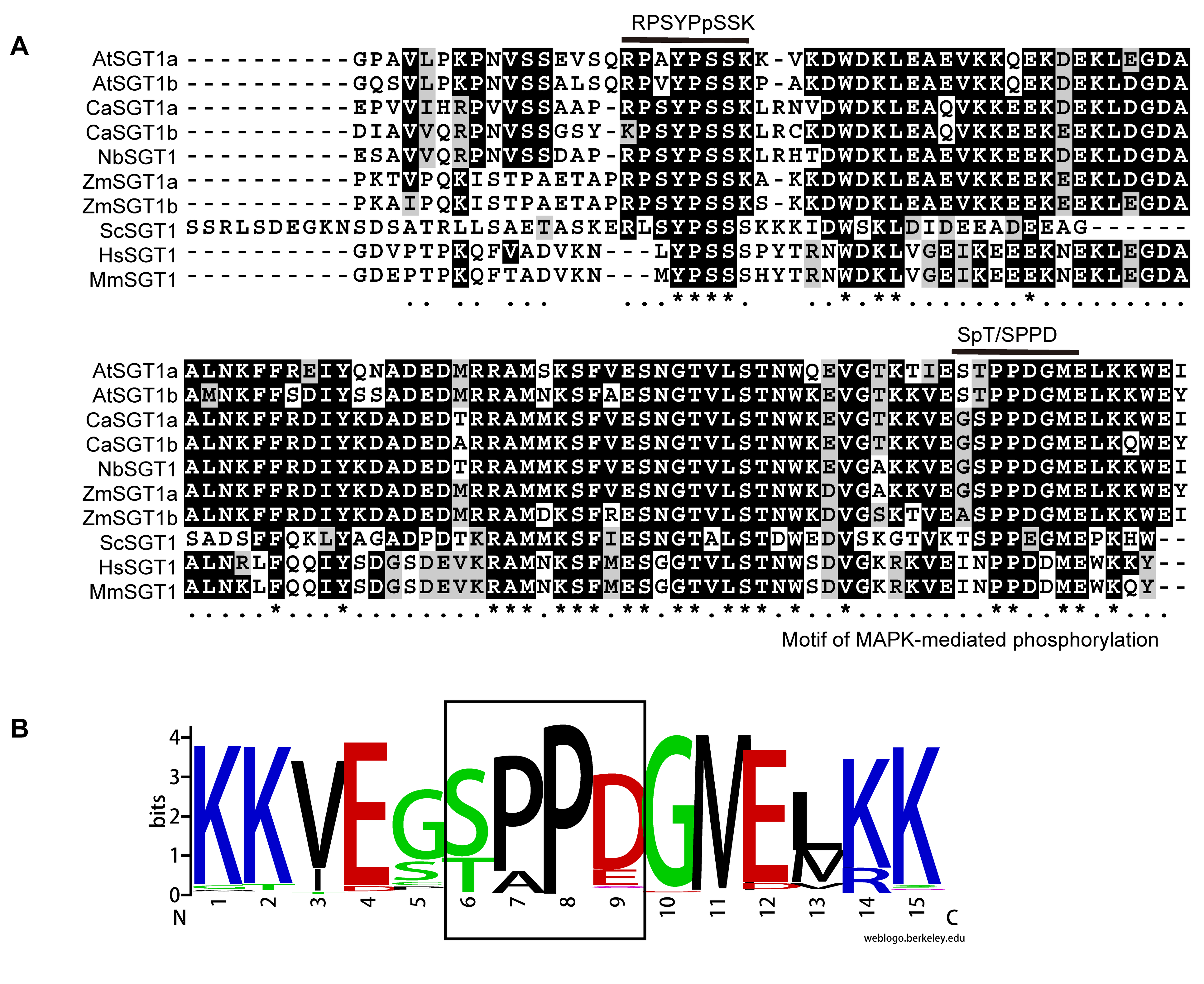

Supplement: S8 Fig — (A) Phosphorylation of S282 and S358 in NbSGT1 are located in the SGS (SGT1-specific) domain. The S358 is predicted to be a MAPK phosphorylation site. At = Arabidopsis thaliana; Ca = Capsicum annuum; Nb = Nicotiana benthamiana; Zm = Zea mays; Sc = Saccharomyces cerevisiae; Hs = Homo sapiens; Ms = Mus musculus. (B) MAPK-mediated phosphorylation motif in SGT1 proteins is conserved in the plant kingdom. SGT1 protein sequences from different plant species genome were retrieved from Phytozome (https://phytozome.jgi.doe.gov/pz/portal.html). Then the sequences were aligned with MEGA program and the MAPK-mediated phosphorylation motif “S/TP” were selected for Weblogo. (TIF) [file ppat.1008933.s008.tif]

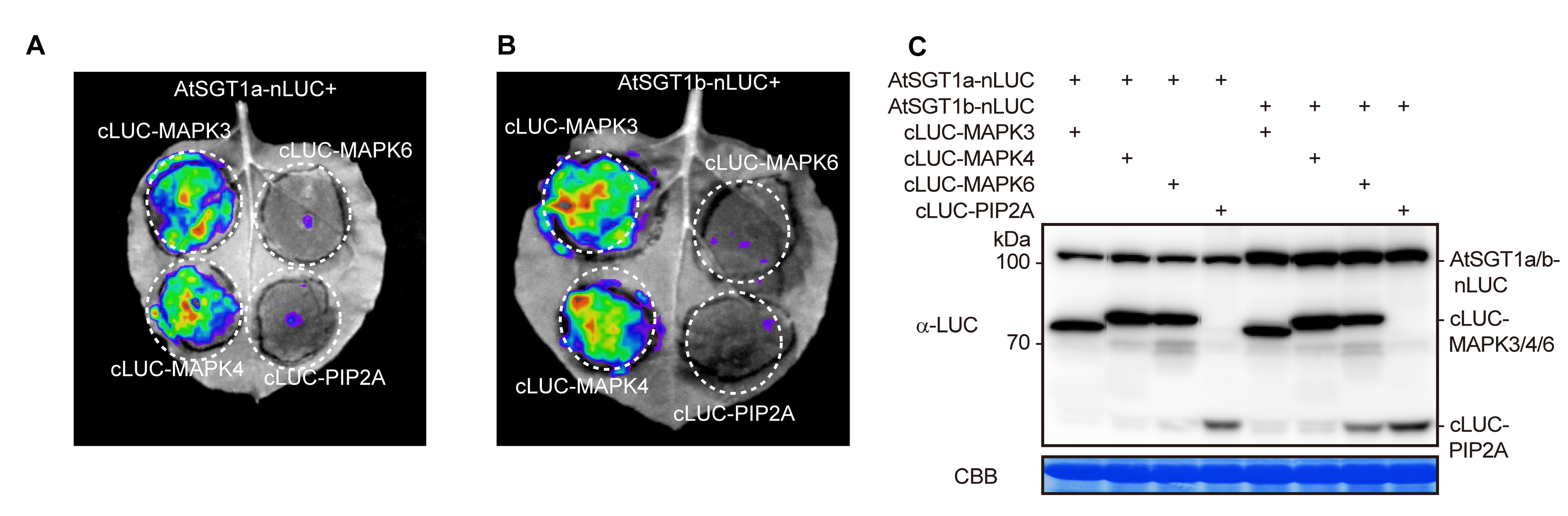

Supplement: S9 Fig — (A, B) AtSGT1a/b associate with AtMAPK3/4, but not with MAPK6, in Split-LUC assay in Nicotiana benthamiana. Agrobacterium combinations with different constructs were infiltrated in N. benthamiana leaves and luciferase activities were examined with CCD imaging machine. The MAPK-PIP2A combination was used as negative control. (C) Protein accumulation in (A) and (B). These experiments were repeated at least 3 times with similar results. In western blot assays, protein marker sizes are provided for reference. (TIF) [file ppat.1008933.s009.tif]

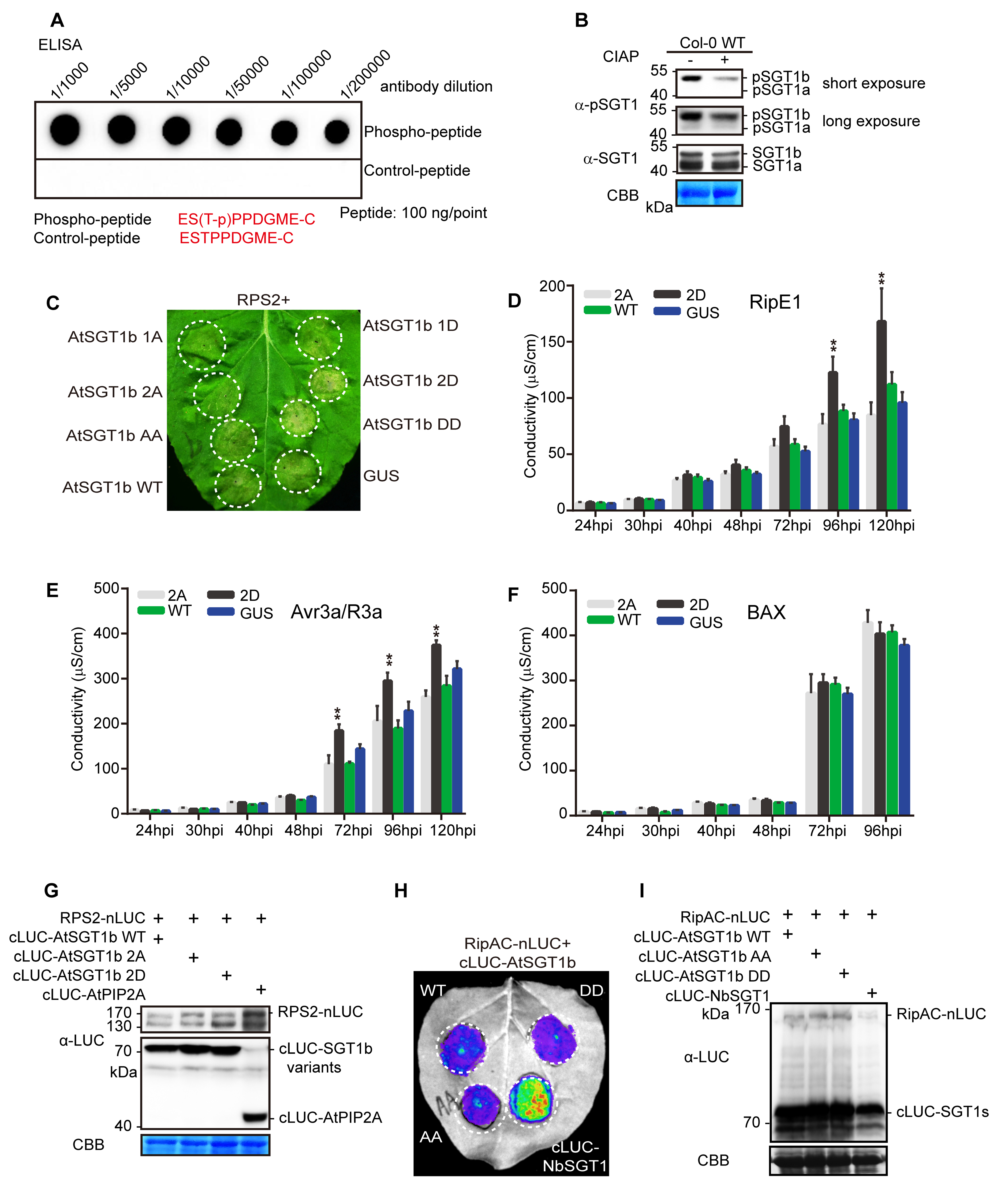

Supplement: S10 Fig — (A) Determination of the specificity of the pSGT1 antibody using an in vitro ELISA assay. (B) Determination of the specificity of pSGT1 antibody in Arabidopsis using CIAP treatment. Two 12-d-old Col-0 WT plants were used for protein extract and CIAP enzyme treatment (37°C, 60min) and the treated protein sample was subjected to western blot with the indicated antibodies. (C) A phospho-mimic mutation in AtSGT1b T346 (T346D) promotes cell death triggered by RPS2 overexpression in N. benthamiana. The experiments were performed as in Fig 5A and the cell death phenotype was recorded 4 dpi with Agrobacterium expressing RPS2. (D-F) A phospho-mimic mutation in AtSGT1b T346 (T346D) promotes cell death triggered by RipE1, Avr3a/R3a, but not BAX overexpression in Nicotiana benthamiana. Agrobacterium expressing AtSGT1b variants or the GUS-FLAG control (OD600 = 0.5) were infiltrated into N. benthamiana leaves 1 day before infiltration with Agrobacterium expressing RipE1, Avr3a/R3a, or BAX (OD600 = 0.15). Leaf discs were taken 21 hpi for conductivity measurements at the indicated time points. The time points in the x-axis are indicated as hpi with Agrobacterium expressing RipE1, Avr3a/R3a, or BAX (mean ± SEM, n = 3, ** p<0.01, t-test, 3 replicates). (G) Western blot shows protein accumulation in Fig 5D and 5E. (H, I) RipAC associates similarly with AtSGT1b variants. The Split-LUC assays were done the same as in Fig 2B. The protein accumulation of each construct is shown in (I). These experiments were repeated at least 3 times with similar results. In western blot assays, protein marker sizes are provided for reference. (TIF) [file ppat.1008933.s010.tif]

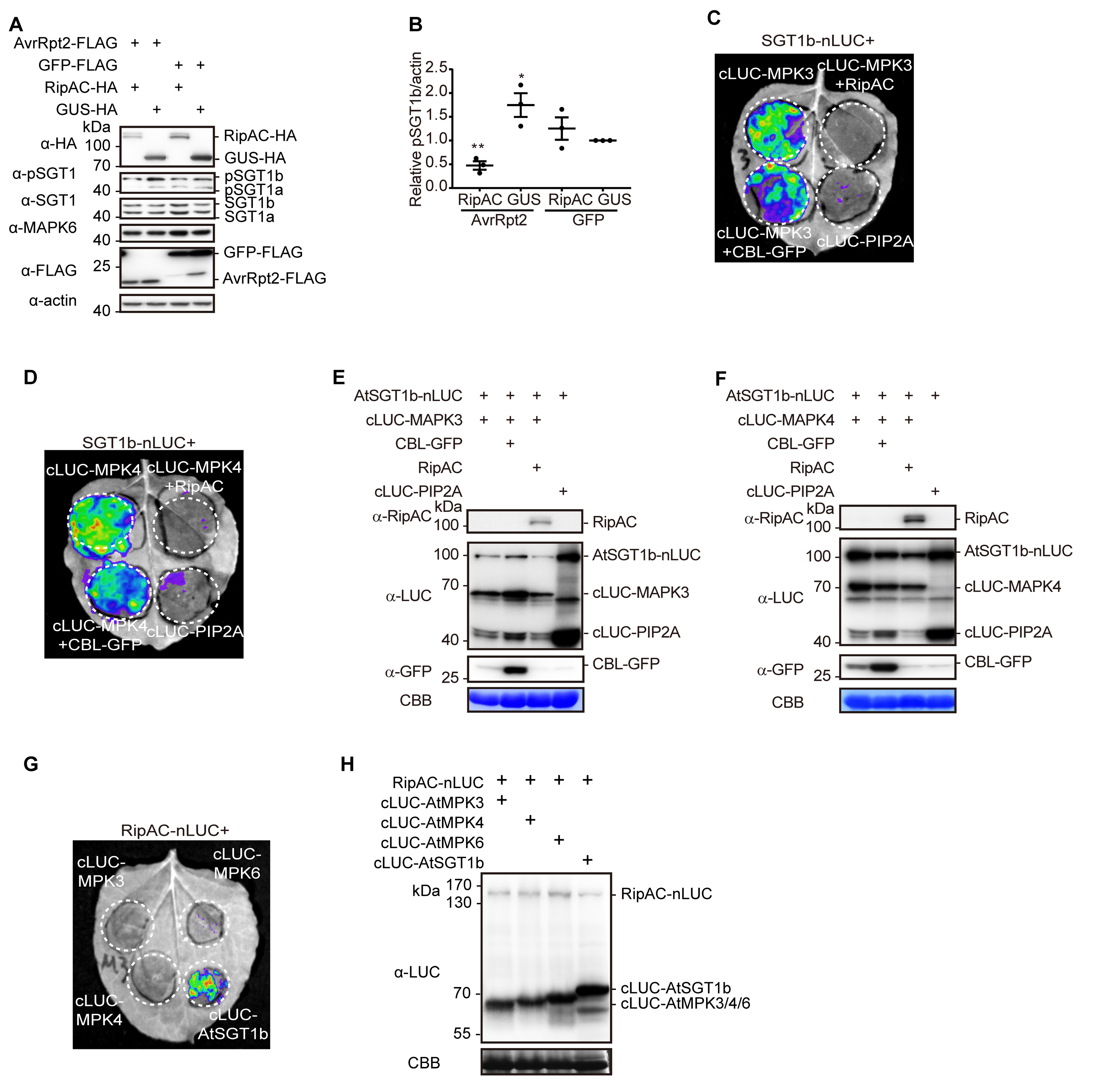

Supplement: S11 Fig — (A) RipAC suppresses ETI-triggered SGT1 phosphorylation in Arabidopsis protoplasts. Western blots were performed as in Fig 4A using samples from protoplasts 10 hours after transfection with the indicated constructs. (B) Quantification of pSGT1b signal normalized to actin and relative to the control sample transfected with GUS and GFP in (A) (mean ± SEM of 3 independent biological replicates, * p<0.05, ** p<0.01, t-test). (C, D) Competitive Split-LUC assays showing that RipAC interferes with the interaction between MAPK3 (C) / MAPK4 (D) and AtSGT1b in Nicotiana benthamiana. Luciferase activity was determined with CCD camera. (E-F) Western blots shows protein accumulation in Fig 6G and 6H, S11C and S11D Fig. (G-H) RipAC does not associate with MPK3/4/6. The Split-LUC assay was performed as in Fig 2B and the RipAC-nLUC/cLUC-AtSGT1b pair was used as positive control. Protein accumulation was determined by western blot as shown in (H). These experiments were repeated at least 3 times with similar results. In western blot assays, protein marker sizes are provided for reference. (TIF) [file ppat.1008933.s011.tif]

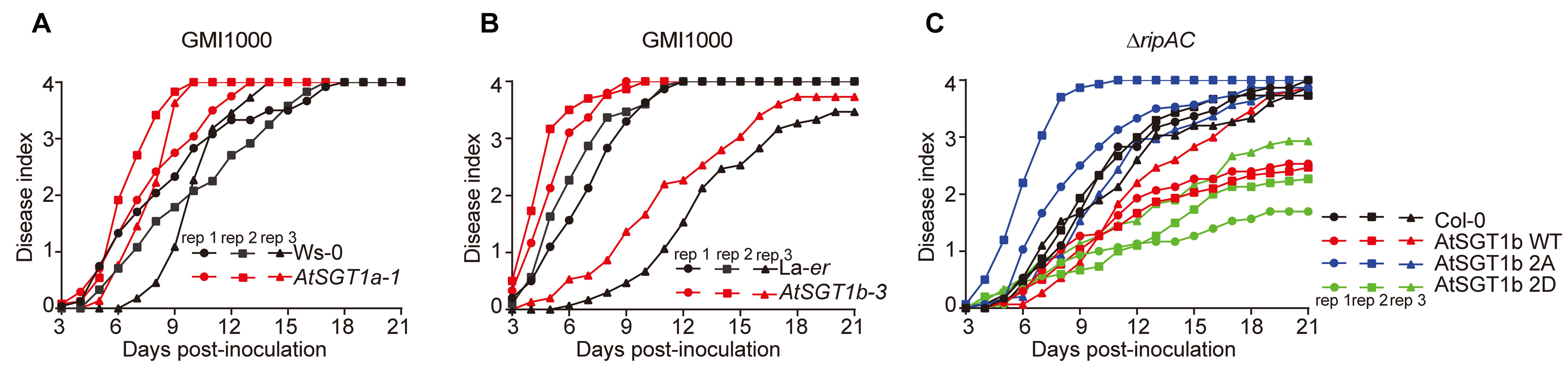

Supplement: S12 Fig — (A, B) Soil-drenching inoculation assays in different Arabidopsis genotypes (AtSGT1a-1 and its wild-type control Ws-0, AtSGT1b-3 and its wild-type control La-er) were performed with GMI1000 WT strain. Composite data from 3 independent biological repeats (average values are shown in Fig 7A and 7B). n = 12 plants per genotype in each repeat in (A) and n = 15 plants per genotype in each repeat in (B). (C) Soil-drenching inoculation assays in Arabidopsis transgenic lines overexpressing AtSGT1b variants were performed with GMI1000 ΔripAC mutant. Composite data from 3 independent biological repeats. n = 15 plants per genotype in each repeat. (TIF) [file ppat.1008933.s012.tif]
