# Supplementary material for: A bacterial effector protein prevents MAPK-mediated phosphorylation of SGT1 to suppress plant immunity
Source: PLoS Pathog. 2020 Sep 25;16(9):e1008933. doi: 10.1371/journal.ppat.1008933 (PMC7540872; doi:10.1371/journal.ppat.1008933)
Supplement: S1 Table — (DOCX) [file ppat.1008933.s013.docx]

**S1 Table. Resources used in this article**

| REAGENT or RESOURCE | SOURCE | IDENTIFIER |
| --- | --- | --- |
| Antibodies | | |
| Mouse monoclonal anti-GFP | Abiocode | Cat# M0802-3a |
| Mouse monoclonal anti-FLAG | Abmart | Cat# M20008 |
| Rabbit polyclonal anti-FLAG | Sigma | Cat# F7425 |
| Rabbit polyclonal anti-Luciferase | Sigma | Cat# L0159 |
| Phospho-p44/42 MAPK (Erk1/2) (Thr202/Tyr204)(20G11) Rabbit mAb antibody | Cell Signaling | Cat# 4370 |
| Mouse monoclonal anti-HA | Roche | Cat# 12CA5 |
| Rabbit polyclonal anti-MAPK6 | Agrisera | Cat# AS12 2633 |
| Rabbit polyclonal anti-actin | Agrisera | Cat# AS13 2640 |
| Rabbit polyclonal anti-AtSGT1a | [1] | N/A |
| Rabbit polyclonal anti-RipAC | This work | N/A |
| Rabbit polyclonal anti-SGT1 pT346 | This work | N/A |
| Rabbit polyclonal anti-H^+^-ATPase | Agrisera | Cat# AS07 260 |
| anti-Mouse IgG-Peroxidase | Sigma | Cat# A2554 |
| anti-Rabbit IgG-Peroxidase | Sigma | Cat# A0545 |
| Bacterial and Virus Strains | | |
| *Escherichia coli* DH5a | Transgen | CD501-3 |
| *Agrobacterium tumefaciens* GV3101 | Weidi Bio | AC1001 |
| *Ralstonia solanacearum* GMI1000 | [2] | N/A |
| *Ralstonia solanacearum* GMI1000 *ΔripAC* | This work | N/A |
| *Ralstonia solanacearum* GMI1000 *ripAC^+^* | This work | N/A |
| *Pseudomonas syringae* pv. *tomato* (Pto)  DC3000 EV | [3] | N/A |
| *Pseudomonas syringae* pv tomato (Pto)  DC3000 *hrcC^-^* | [4] |  |
| *Pseudomonas syringae* pv. *tomato* (Pto)  DC3000 AvrRpm1 | [3] | N/A |
| *Pseudomonas syringae* pv. *tomato* (Pto)  DC3000 AvrRpt2 | [3] | N/A |
| *Pseudomonas syringae* pv. *tomato* (Pto)  DC3000 AvrRps4 | [3] | N/A |
| Chemicals, Peptides, and Recombinant Proteins | | |
| Protease Inhibitor Cocktail for plant cell and tissue extracts, DMSO solution | Sigma | P9599 |
| GFP-Trap_A | Chromotek | Cat# gta-100 |
| ANTI-FLAG M2 Affinity Gel | Sigma-Aldrich | Cat# A2220 |
| XenoLight D-Luciferin | PerkinElmer | Cat# 122799 |
| Critical Commercial Assays |  |  |
| pENTR/D-TOPO Cloning Kit | Invitrogen | Cat# K240020SP |
| Gateway LR Clonase II Enzyme Mix | Invitrogen | Cat# 11791100 |
| Calf Intestinal Alkaline Phosphatase | New England Lab | Cat# NEB#M0290 |
| Glutathione Sepharose 4 Fast Flow | GE Healthcare | Cat#17-5132-01 |
| Ni-NTA His Bind Resin | Novagen | Cat# 70666-4 |
| Experimental Models: Organisms/Strains | | |
| Arabidopsis: 35S:RipAC-GFP (AC#3 and AC #31) | This work | N/A |
| Arabidopsis: GVG:AvrRpt2 | [5] | N/A |
| Arabidopsis: GVG:MKK5DD | [6] | N/A |
| Arabidopsis: MPK3SR28 (*mpk3mpk6* P_MPK3_:*MPK3* TG, line #28) | [7] | N/A |
| Arabidopsis: MPK6SR58 (*mpk3mpk6* P_MPK6_:*MPK6* YG, line #58) | [8] | N/A |
| Arabidopsis: *AtSGT1a-1* (Ws-0) | [9] | N/A |
| Arabidopsis: *AtSGT1b-3* (La-er) | [10] | N/A |
| Arabidopsis: GVG:AvrRpt2/Col-0 | This work | N/A |
| Arabidopsis: GVG:AvrRpt2/AC #3 | This work | N/A |
| Arabidopsis: 35S:AtSGT1b WT | This work | N/A |
| Arabidopsis: 35S:AtSGT1b T346A (2A) | This work | N/A |
| Arabidopsis: 35S:AtSGT1b T346D (2D) | This work | N/A |
| *Solanum lycopersicum* cv. Moneymaker | N/A | N/A |
| Oligonucleotides | | |
| Primers see Table S2 | Ruidi Biotech | Custom order |
| Recombinant DNA | | |
| pENTR/D-TOPO | ThermoFisher | Cat# K240020 |
| pGWB502 | [11] | N/A |
| pGWB505 | [11] | N/A |
| pGWB511 | [11] | N/A |
| pGWB554 | [11] | N/A |
| pGTQL1211YN | [12] | Addgene # 61704 |
| pGTQL1221YC | [12] | Addgene # 61705 |
| pGWB-nLUC | [13] | N/A |
| pGWB-cLUC | This work | N/A |
| pEASYBLUNT-LB-Gm-RB | This work | N/A |
| pRCT-pRipAC-RipAC | This work | N/A |
| pGWB502-RipAC (no tag) | This work | N/A |
| pGWB505-RipAC-GFP | This work | N/A |
| pGWB-RipAC-nLUC | This work | N/A |
| pGWB-cLUC-RipAC | This work | N/A |
| pGWB502-CBL-GFP (no tag) | This work | N/A |
| 35S:RPS2-HA | [14] | N/A |
| 35S:R3a | [15] | N/A |
| 35S:Avr3a | [15] | N/A |
| 35S:Bax | [15] | N/A |
| 35S:INF1 | [15] | N/A |
| pGWB511-AtSGT1a-FLAG | This work | N/A |
| pGWB511-AtSGT1b-FLAG | This work | N/A |
| pGWB511-NbSGT1-FLAG | This work | N/A |
| pGWB511-SlSGT1b-FLAG | This work | N/A |
| pGWB554-AtSGT1a-RFP | This work | N/A |
| pGWB554-AtSGT1b-RFP | This work | N/A |
| pGWB554-NbSGT1-RFP | This work | N/A |
| pGWB-AtSGT1a-nLUC | This work | N/A |
| pGWB-AtSGT1b-nLUC | This work | N/A |
| pGWB-cLUC-AtSGT1a | This work | N/A |
| pGWB-cLUC-AtSGT1b | This work | N/A |
| pGWB-cLUC-NbSGT1 | This work | N/A |
| pGWB-cLUC-SlSGT1b | This work | N/A |
| pGWB-cLUC-AtPIP2A | This work | N/A |
| pCAMBIA-AtFLS2-nLUC | [16] | N/A |
| pGWB512-FLAG-AtMAPK3 | This work | N/A |
| pGWB512-FLAG-AtMAPK4 | This work | N/A |
| pGWB512-FLAG-AtMAPK6 | This work | N/A |
| pGWB-cLUC-AtMAPK3 | This work | N/A |
| pGWB-cLUC-AtMAPK4 | This work | N/A |
| pGWB-cLUC-AtMAPK6 | This work | N/A |
| pGWB511-GUS-FLAG | [1] | N/A |
| pGTQL1211-RipAC-nYFP | This work | N/A |
| pGTQL1211-RipAC-cYFP | This work | N/A |
| pGTQL1211-AtPIP2A-nYFP | This work | N/A |
| pGTQL1211-AtPIP2A-cYFP | This work | N/A |
| pGTQL1211-AtSGT1a-cYFP | This work | N/A |
| pGTQL1211-AtSGT1b-cYFP | This work | N/A |
| pGTQL1211-NbSGT1-cYFP | This work | N/A |
| pGWB511-AtSGT1b TPR-FLAG | This work | N/A |
| pGWB511-AtSGT1b TPR+CS-FLAG | This work | N/A |
| pGWB511-AtSGT1b CS-FLAG | This work | N/A |
| pGWB511-AtSGT1b CS+SGS-FLAG | This work | N/A |
| pGWB511-AtSGT1b SGS-FLAG | This work | N/A |
| pCAMBIA-RPS2-nLUC | This work | N/A |
| pGWB502-AtSGT1b S271A (no tag) | This work | N/A |
| pGWB502-AtSGT1b S271D (no tag) | This work | N/A |
| pGWB502-AtSGT1b T346A (no tag) | This work | N/A |
| pGWB502-AtSGT1b T346D (no tag) | This work | N/A |
| pGWB502-AtSGT1b S271AT346A (no tag) | This work | N/A |
| pGWB502-AtSGT1b S271D T346D (no tag) | This work | N/A |
| pGWB-cLUC-AtSGT1b S271A | This work | N/A |
| pGWB-cLUC-AtSGT1b S271D | This work | N/A |
| pGWB-cLUC-AtSGT1b T346A | This work | N/A |
| pGWB-cLUC-AtSGT1b T346D | This work | N/A |
| pGWB-cLUC-AtSGT1b S271AT346A | This work | N/A |
| pGWB-cLUC-AtSGT1b S271D T346D | This work | N/A |
| pGWB-505-GFP | This work | N/A |
| pGWB-505-AtSGT1b-GFP | This work | N/A |
| pGWB-505-AtSGT1b S271AT346A-GFP | This work | N/A |
| pGWB-505-AtSGT1b S271D T346D-GFP | This work | N/A |
| pGWB505-RipE1 | [17] | N/A |
| pGWB-cLUC-AtPIP2A | This work | N/A |
| pHBT-AvrRpt2-FLAG | This work | N/A |
| pHBT-GFP-FLAG | This work | N/A |
| pXCSG-RipAC-HA-Strep | This work | N/A |
| pXCSG-GUS-HA-Strep | This work | N/A |
| His-SUMO-RipAC | This work | N/A |
| pET28-AtMPK3 | [18] | N/A |
| pET28-AtMPK6 | [18] | N/A |
| pET28-AtMKK5DD | [18] | N/A |
| pGEX-6p-1-AtSGT1a | [19] | N/A |
| pGEX-6p-1-AtSGT1b | [19] | N/A |
| pGEX-6p-1-GUS | [19] | N/A |
| Software and Algorithms |  |  |
| Prism 7 | GraphPad Software | https://www.graphpad.com/scientific-software/prism/ |
| Scaffold 4.0 | Proteome Software | http://www.proteomesoftware.com/products/scaffold/ |
| Adobe Illustrator CS6 (64Bit) | Adobe Illustrator Software | https://www.adobe.com/products/illustrator.html |
| ImageJ | NIH ImageJ | https://imagej.nih.gov/ij/ |

**References**

1. Yu G, Xian L, Sang Y, Macho AP (2019) Cautionary notes on the use of Agrobacterium-mediated transient gene expression upon SGT1 silencing in *Nicotiana benthamiana*. New Phytologist 222: 14-17.

2. Salanoubat M, Genin S, Artiguenave F, Gouzy J, Mangenot S, et al. (2002) Genome sequence of the plant pathogen *Ralstonia solanacearum*. Nature 415: 497-502.

3. Macho AP, Guevara CM, Tornero P, Ruiz-Albert J, Beuzón CR (2010) The *Pseudomonas syringae* effector protein HopZ1a suppresses effector-triggered immunity. New Phytologist 187: 1018-1033.

4. Ronald PC, Salmeron JM, Carland FM, Staskawicz BJ (1992) The cloned avirulence gene *avrPto* induces disease resistance in tomato cultivars containing the *Pto* resistance gene. Journal of bacteriology 174: 1604-1611.

5. McNellis TW, Mudgett MB, Li K, Aoyama T, Horvath D, et al. (1998) Glucocorticoid-inducible expression of a bacterial avirulence gene in transgenic Arabidopsis induces hypersensitive cell death. The Plant Journal 14: 247-257.

6. Ren D, Yang H, Zhang S (2002) Cell death mediated by MAPK is associated with hydrogen peroxide production in Arabidopsis. Journal of Biological Chemistry 277: 559-565.

7. Su J, Zhang M, Zhang L, Sun T, Liu Y, et al. (2017) Regulation of stomatal immunity by interdependent functions of a pathogen-responsive MPK3/MPK6 cascade and abscisic acid. The Plant Cell 29: 526-542.

8. Xu J, Xie J, Yan C, Zou X, Ren D, et al. (2014) A chemical genetic approach demonstrates that MPK3/MPK6 activation and NADPH oxidase-mediated oxidative burst are two independent signaling events in plant immunity. The Plant Journal 77: 222-234.

9. Azevedo C, Betsuyaku S, Peart J, Takahashi A, Noël L, et al. (2006) Role of SGT1 in resistance protein accumulation in plant immunity. The EMBO Journal 25: 2007-2016.

10. Austin MJ, Muskett P, Kahn K, Feys BJ, Jones JDG, et al. (2002) Regulatory role of SGT1 in early R gene-mediated plant defenses. Science 295: 2077-2080.

11. Nakagawa T, Suzuki T, Murata S, Nakamura S, Hino T, et al. (2007) Improved gateway binary vectors: high-performance vectors for creation of fusion constructs in transgenic analysis of plants. Bioscience, Biotechnology, and Biochemistry 71: 2095-2100.

12. Lu Q, Tang X, Tian G, Wang F, Liu K, et al. (2010) Arabidopsis homolog of the yeast TREX-2 mRNA export complex: components and anchoring nucleoporin. The Plant Journal 61: 259-270.

13. Wang Y, Li Y, Rosas-Diaz T, Caceres-Moreno C, Lozano-Durán R, et al. (2019) The IMMUNE-ASSOCIATED NUCLEOTIDE-BINDING 9 protein is a regulator of basal immunity in *Arabidopsis thaliana*. Molecular Plant-Microbe Interactions 32: 65-75.

14. Li M, Ma X, Chiang Y-H, Yadeta KA, Ding P, et al. (2014) Proline isomerization of the immune receptor-interacting protein RIN4 by a cyclophilin inhibits effector-triggered immunity in Arabidopsis. Cell host & microbe 16: 473-483.

15. Liu T, Ye W, Ru Y, Yang X, Gu B, et al. (2011) Two host cytoplasmic effectors are required for pathogenesis of *Phytophthora sojae* by suppression of host defenses. Plant Physiology 155: 490-501.

16. Li L, Li M, Yu L, Zhou Z, Liang X, et al. (2014) The FLS2-associated kinase BIK1 directly phosphorylates the NADPH oxidase RbohD to control plant immunity. Cell host & microbe 15: 329-338.

17. Sang Y, Yu W, Zhuang H, Wei Y, Derevnina L, et al. (2020) Intra-strain elicitation and suppression of plant immunity by *Ralstonia solanacearum* type-III effectors in *Nicotiana benthamiana*. Plant Communications https://doi.org/10.1016/j.xplc.2020.100025: 100025.

18. Zhao C, Wang P, Si T, Hsu C-C, Wang L, et al. (2017) MAP kinase cascades regulate the cold response by modulating ICE1 protein stability. Developmental Cell 45: 618-629.e615.

19. Botër M, Amigues B, Peart J, Breuer C, Kadota Y, et al. (2007) Structural and functional analysis of SGT1 reveals that its interaction with HSP90 is required for the accumulation of Rx, an R protein involved in plant immunity. The Plant Cell 19: 3791-3804.
