# Supplementary material for: A bacterial effector protein prevents MAPK-mediated phosphorylation of SGT1 to suppress plant immunity
Source: PLoS Pathog. 2020 Sep 25;16(9):e1008933. doi: 10.1371/journal.ppat.1008933 (PMC7540872; doi:10.1371/journal.ppat.1008933)
Supplement: S2 Table — (DOCX) [file ppat.1008933.s014.docx]

**S2 Table. Primers used in this study.**

| Name | Sequence 5’ to 3’ | Purpose |
| --- | --- | --- |
| RipAC-LB-F | CGATTTCACGCCGGCGAAGAC | Clone *RipAC* left board |
| RipAC-LB-R | CCGGATCAAGAATTCGAGCGTCAGGCTAAGTCGCG |  |
| RipAC-RB-F | CCTGACGCTCGAATTCTTGATCCGGTGCCGCATCCC | Clone *RipAC* right board |
| RipAC-RB-R | CGACCAGGCGCGCATCACC |  |
| pRipAC-F | CCCCCTAGGTCACTTATTGGAAACTCCCGTC | Clone *RipABC* operon promoter |
| pRipAC-R | GGGGTACCCAGGCTCCGGCGGGCCGGAGCGC |  |
| RipAC-F | CACCATGCCTATCCTTCCACGCCTATTCCATCGA | Clone *RipAC* CDS |
| RipAC-R | ACGCTGCCTCGACGGACTTGCCGCGGGCGT |  |
| AtSGT1a-F | CACCATGGCGAAGGAGCTTGCT | Clone *AtSGT1a* CDS |
| AtSGT1a-R | GATCTCCCATTTCTTGAGCTCCAT |  |
| AtSGT1b-F | CACCATGGCCAAGGAATTAGCAGAGAAAGCTAAAGAAGCT | Clone *AtSGT1b* CDS |
| AtSGT1b-R | ATACTCCCACTTCTTGAGCTCCATGCCATCTG |  |
| NbSGT1-F | CACCATGGCGTCCGATCTGGAGATTAGGGC | Clone *NbSGT1* CDS |
| NbSGT1-R | GATTTCCCATTTCTTCAGCTCCATGCC |  |
| SlSGT1b-F | CACCATGGCGTCCGATCTGGAGACTAGGGCTAAA | Clone *SlSGT1b* CDS |
| SlSGT1b -R | GATCTCCCATTTCTTCAGCTCCATGCC |  |
| AtPIP2A-F | CACCATGGCAAAGGATGTGGAAGCCGTTCCCG | Clone *AtPIP2A* CDS |
| AtPIP2A-R | GACGTTGGCAGCACTTCTGAATGATCCG |  |
| CBL-GFP-F | CACCATGGGCTGCTTCCACTCAAAGGCAGCAAAAGAATTTATGGTGAGCAAGGGCGAGGAGCTGTTCA | Clone *CBL-GFP* CDS |
| GFP-R | CTTGTACAGCTCGTCCATGCCGTGAGTG |  |
| AvrRpt2-F | ATGAAAATTGCTCCAGTTGCCAT | RT-PCR |
| AvrRpt2-R | GTAGAGCATTGCGTGTGGAAC | RT-PCR |
| Atactin2-F | TGCTGGACGTGACCTTACTG | RT-PCR |
| Atactin2-R | TTCTCGATGGAAGAGCTGGT | RT-PCR |
| AtSGT1b S271A-F | ttttgctggcttagaagctgggtacactggtctct | Generate *AtSGT1b* mutant (S271A) |
| AtSGT1b S271A-R | agagaccagtgtacccagcttctaagccagcaaaa |  |
| AtSGT1b S271D-F | gtcttttgctggcttagaatctgggtacactggtctctgc | Generate *AtSGT1b* mutant (S271D) |
| AtSGT1b S271D-R | gcagagaccagtgtacccagattctaagccagcaaaagac |  |
| AtSGT1b T346A-F | catgccatctggtggagcgctctccactttcttag | Generate *AtSGT1b* mutant (T346A) |
| AtSGT1b T346A-R | ctaagaaagtggagagcgctccaccagatggcatg |  |
| AtSGT1b T346D-F | ctccatgccatctggtggatcgctctccactttcttagtc | Generate *AtSGT1b* mutant (T346D) |
| AtSGT1b T346D-R | gactaagaaagtggagagcgatccaccagatggcatggag |  |
| NbSGT1 TPR-R | GGCAGCAGATCCTTGATAGGACAG | Generate *NbSGT1* TPR truncation |
| NbSGT1 CS-F | CACCATGTCTGAGTCTTTGGGCAATGTTGCTG | Generate *NbSGT1* CS truncation |
| NbSGT1 CS-R | AGACTCTCTCGTATATTCGAGAGATG |  |
| NbSGT1 SGS-F | CACCATGTATACGAGAGAGTCTGCTGTAGTGC | Generate *NbSGT1* SGS truncation |
| AtMAPK4-F | CACCATGTCGGCGGAGAGTTGTTTCGGAAGCTCG | Clone *AtMAPK4* CDS |
| AtMAPK4-R | CACTGAGTCTTGAGGATTGAACTTGACT |  |
| pHBT-AvrRpt2- FLAG-F | CTTGCTCCGTGGATCCTCTAGAATGAAAATTGCTCCAGTTGCC | Clone AvrRpt2 into pHBT-35S-FLAG |
| pHBT-AvrRpt2- FLAG-R | TGTAGTCAGAAGGCCTGGTACCGCGGTAGAGCATTGCGTGTG |  |
| pHBT-GFP- FLAG-F | CTTGCTCCGTGGATCCTCTAGAATGGTGAGCAAGGGCGA | Clone GFP into pHBT-35S-FLAG |
| pHBT-GFP- FLAG-R | TGTAGTCAGAAGGCCTGGTACCCTTGTACAGCTCGTCCATG |  |
| RPS2-nLUC-F | gagaacacgggggacgagctcATGGATTTCATCTCATCTCTTATCG | Clone RPS CDS into pCAMBIA-nLUC |
| RPS2-nLUC-R | cgcgtacgagatctggtcgacATTTGGAACAAAGCGCGGT |  |
| His-SUMO-RipAC-F | CACAGAGAACAGATTGGTATGCCTATCCTTCCACGCCTATTC | Clone RipAC CDS into His-SUMO |
| His-SUMO-RipAC-R | GTTCGACTTAAGCATTATTCAACGCTGCCTCGACGGACTTGC |  |
