# Supplementary material for: A bacterial effector protein prevents MAPK-mediated phosphorylation of SGT1 to suppress plant immunity
Source: PLoS Pathog. 2020 Sep 25;16(9):e1008933. doi: 10.1371/journal.ppat.1008933 (PMC7540872; doi:10.1371/journal.ppat.1008933)
Supplement: Attachment — Submitted filename: Response to reviewers-Final.docx [file ppat.1008933.s015.docx]

**Dear editor,**

**We would like to thank you for offering us the opportunity to prepare a revised version of our manuscript. We would also like to thank the reviewers for their positive feedback and constructive suggestions. We have addressed all their concerns and modified our manuscript accordingly, and indeed we believe that our manuscript is much improved as a result. Please find attached a point-by-point response to the reviewers’ comments (our responses are in bold blue text). We are submitting a revised manuscript with tracked changes and a “clean” version, with all the changes accepted, as requested in the re-submission guidelines.**

**Sincerely,**

**Alberto Macho (on behalf of all authors)**

------

Reviewer's Responses to Questions

**Part I - Summary**
Please use this section to discuss strengths/weaknesses of study, novelty/significance, general execution and scholarship.

Reviewer #1: In this manuscript, the authors identified “A bacterial effector protein prevents MAPK-mediated phosphorylation of SGT1 to suppress plant immunity”. The story is of sufficient interest and novelty to warrant publication in PLoS Pathogens in my opinion. The authors showed a nice example that how plant pathogen Ralstonia solanacearum effector RipAC inhibits the interaction between SGT1 and MAP kinases, as well as the phosphorylation of a MAPK target motif in the C-terminal domain of SGT1. The presentation that RipAC suppresses ETI to manipulate plant immunity by targeting SGT1 in this manuscript is sorted in a clear logic. However, there are still some concerns need to be addressed before it can be accepted by the journal.

Reviewer #2: The NLR mediated immune response is important for recognizing pathogen effectors. The NLR resistance proteins, such as PRS2, Prf, Rx, RPS5 and RPM1…., can form a complex with other proteins (e.g. RAR1, require for Mla12 resistance; HSP90, heat shock protein 90; and SGT1, suppressor of the G2 allele of skp1) to regulate plant resistance by R protein accumulation and/or activation. Among these three proteins, several studies showed that bacterial effector (AvrBsT) and fungi effector (See1) can target SGT1 to regulate avrBsT-triggered cell death and DNA synthesis. In this study, the authors demonstrate that another effector from Ralstonia solanacearum, RipAC, also associates with SGT1 to regulate NLR (RPS2)-mediated immunity. The authors first showed that RipAC contributed to the virulence of R. solanacearum, then they further identified SGT1 as its target via MS analysis. In addition, SGT1 can associate with MPK4/6 and be phosphorylated by MAP kinases at two serine residues (NbSGT1 S282 and S358). RipAC reduced SGT1 phosphorylation via reducing the association of SGT1 and MPK6. Furthermore, SGT1 phosphorylation is required for RPS2-triggered cell death in N. benthamiana and RipAC virulence function in Arabidopsis. Overall, the work showed clear data that SGT1 is a target of RipAC and the virulence function of RipAC requires the phosphorylation of SGT1. Moreover, the manuscript is easy to follow and logic is clear. But I feel that some data do not directly support the conclusion, especially the part of SGT1 phosphorylation depends on MPK kinase (please see the comment 4). Also, the mechanism of SGT1 regulating NLR-mediated immunity is different, such as SGT1 stabilized NLR Rx and Prf (Azevedo et al, 2006, EMBO journal; Kud et al, 2013, Bio.&Bio Res. Commun.), but also destabilized RPS5 and RPM1 (Holt III et al, 2005, Science), so I think the authors should turn down some conclusions, like phosphorylation of SGT1 contributes to the activation of NLR-mediated responses in line 28-29. It might be better to use “RPS2-mediated response” instead of “NLR-mediated responses” when the authors make a conclusion.

**>>Response: We have modified that sentence accordingly.**

I think it will be worth considering to publish this study in Plos Pathogens if the authors can clarify the following questions.
1. The authors claimed that RipAC contributed to R. solanacearum infection in Arabidopsis expressing RipAC in Figure 1D, but not Pto in Figure 3G. Do the authors think that the plant defense suppressed by RipAC is general or specific?

**>>Response: This is an interesting observation. Indeed we detected enhanced susceptibility of the RipAC lines against Ralstonia, but not Pto DC3000. This may suggest that RipAC is able to suppress basal immunity against Ralstonia, but not Pto DC3000, since basal immunity against these pathogens may have different requirements in terms of specific immune components. Besides its suppression of NLR-mediated immunity (shown in this manuscript), we know that RipAC is also able to suppress PRR-mediated immunity (manuscript in preparation) through the targeting of different immune components; in that project, we performed specific assays to study in detail the suppression of PRR-mediated immunity. Therefore, we think that RipAC has several different specific activities to suppress immunity. Another reason for these different results may be the different inoculation methods, since Ralstonia was inoculated by soil-drenching, while Pto DC3000 was inoculated by direct infiltration inside leaf tissues. These conditions may not be ideal to detect differences in basal immunity against Pto DC3000; however, they allowed us to clearly determine that RipAC lines have compromised immunity against Pto expressing AvrRpm1, AvrRpt2, and AvrRps4.**

2. In Figure 3H-J, expressing RipAC in Arabidopsis enhanced Pto AvrRpm1, Pto AvrRpt2, Pto AvrRps4 infection, how about the Arabidopsis HR phenotype in these leaves triggered by these effectors and R genes recognitioin? The authors should include these data in the figure to see whether RipAC affects HR.

**>>Response: In these experiments we tested whether NLR-mediated immunity is compromised in RipAC-expressing Arabidopsis. We considered that bacterial counts have higher sensitivity than macroscopic HR assays in Arabidopsis, which often do not have enough resolution to detect quantitative contributions. Moreover, the measurement of bacterial growth of Pto in Arabidopsis is particularly accurate and straightforward. For these reasons, we did not perform macroscopic HR assays, which we did not consider a limitation to reach our conclusion, considering that the final outcome of NLR-mediated immunity is the restriction of bacterial growth. We have modified the text to indicate that we specifically observed bacterial growth: “…indicating that RipAC is able to effectively suppress the final outcome of NLR-mediated immunity (i.e. the restriction of bacterial growth) in Arabidopsis.” (Lines 261-264)**

3. co-IP, Split-LUC and split-YFP assays could not demonstrate direct interaction of RipAC with SGT1, just association. If the authors want to suggest directly interaction, in vitro pull-down assay is needed.

**>>Response: We thank the reviewer for this suggestion. We have performed in vitro pull-down assays, and they confirm the direct interaction between RipAC and AtSGT1a/b. The new results are mentioned in the revised manuscript text and shown in the new Figure 2C.**

Also, why the authors did not perform Split-LUC assay for SlSGT1 and RipAC? Considering SlSGT1 is also important for RipAC-mediated infection promotion in tomato, these data should be included.

**>>Response: We have now also validated the interaction between RipAC and SlSGT1 by Split-LUC, and the result is shown in Figure 2 (new Figure 2B).**

4. The data supporting AtSGT1a/b phosphorylation by MPK3/4/6 are not sufficient and not direct in Figure 4D. The authors just used AvrRpt2 to activate MAPK kinase, then test whether AtSGT1a/b phosphorylation are affected. Several experiments could be used: 1) co-expression of SGT1a/b with MPK4, MPK6 (the authors used in Figure 6G) or MPK3 together with MKK5DD (constitutive activation of MPK3/6) or loss-of-function MKK5KR (constitutive inactivation of MPK3/6) (Guo et al, Nat. Comm., 2016) to see whether SGT1a/b phosphorylation was enhanced or reduced. 2) test SGT1a/b phosphorylation in mpk3, mpk4, mpk6 single mutants, or mpk3/mpk6 double mutant (silencing of mpk6 in mpk3 mutant, reported by Yu et al, Cell reports, 2019). 3) in vitro kinase assay for MPK3, MKK9DD and SGT1 as shown in Guo et al (Nat. Comm., 2016) to show that SGT1 is directly phosphorylated by MAP kinase. 4) use a AvrRpt2 mutants that can not induce MAPK activation to test SGT1a/b phosphorylation as a control in Figure 4D.

**>>Response: We thank the reviewer for these suggestions. As suggested by the reviewer, we have undertaken several complementary approaches to validate and strengthen this point. First, using our anti-pSGT1 antibody after *in vitro* kinase assays we observed that recombinant AtMAPK3 and AtMAPK6 directly phosphorylate recombinant AtSGT1a and AtSGT1b, and this phosphorylation was stronger in the presence of AtMKK5DD (new Figure 4D). Second, we used Arabidopsis DEX:MKK5DD plants; we show that MKK5DD expression, which leads to constitutive activation of MPK3/6, enhances SGT1 phosphorylation (Figure 4E and 4F). Third, we used Arabidopsis WT plants and mutants with abolished AtMAPK3 and AtMAPK6 activity in *Atmapk6* and *Atmapk3* mutant backgrounds, respectively (Xu et al, Plant J, 2014; Su et al, Plant Cell, 2017), and inoculated it with Pto AvrRpt2. The enhancement of SGT1 phosphorylation observed in WT plants was not observed in the mpk3/mpk6 loss-of-function plants, confirming that SGT1 phosphorylation requires these MAPKs (Figure 4I and 4J). These results are now shown in Figure 4, and the corresponding text has been included in lines 291-308.**

5. Did the authors test RPS2, Avr3a/R3 and RipE2-induced HR in SGT1-silenced plants in N. benthamiana? Also, how about RipAC suppressed RPS2, Avr3a/R3 and RipE2-induced HR in SGT1-silenced plants in N. benthamiana?

**>>Response: As far as we know, to date, all NLRs seem to depend on SGT1 for function. We did not directly test RPS2-induced HR in SGT1-silenced plants in N. benthamiana, but we have observed that RPS2-mediated immunity is compromised in SGT1-silenced Arabidopsis (Yu et al, 2020;** [**https://doi.org/10.1101/2020.05.08.083766**](https://doi.org/10.1101/2020.05.08.083766)**). In SGT1-silenced plants, neither Avr3a/R3A nor RipE1 trigger HR (Bos et al, 2006;** [**https://doi.org/10.1111/j.1365-313X.2006.02866.x**](https://doi.org/10.1111/j.1365-313X.2006.02866.x)**, Sang et al, 2020;** [**https://doi.org/10.1016/j.xplc.2020.100025**](https://doi.org/10.1016/j.xplc.2020.100025)**). Given the absence of immune responses in SGT1-silenced plants, we did not further perform RipAC expression.**

6. RipAC reduced AtSGT1a/b phosphorylation in Arabidopsis expressing RipAC in Figure 6A. Was AtSGT1a/b phosphorylation affected in Arabidopsis and tomato after infected by R. solanacearum?

**>>Response: We performed infiltration of Arabidopsis leaves with the *R. solanacearum* *ripAC* mutant and observed a slight increase in SGT1 phosphorylation, which was not observed upon inoculation with wild-type *R. solanacearum* GMI1000 (Figures 6E and 6F). This result contributes to the idea that RipAC indeed suppresses SGT1 phosphorylation during *R. solanacearum* infection, and it is now mentioned in the manuscript text (lines 355-358).**

7. The author claimed that RipAC inhibited the SGT1-MPK3/4/6 interaction, resulting in reduced SGT1 phosphorylation in Figure 6G, 6E, 6F. However, in Figures 3C, 3D, 3E, 6C, RipAC also reduced the MPK3/6 phosphorylation. So is it possible that RipAC reduces the phosphorylation of MPK3/4/6 resulting in a decrease in SGT1 phosphorylation? Did the authors test whether RipAC can interact with MPK3/4/6?

**>>Response: We have performed additional experiments to clarify this point. We did not detect interaction between RipAC and MPK3/4/6. These results are now included in Figures S11G-H and mentioned in the main text (lines 365-366). Therefore, we believe that the RipAC-mediated suppression of MPK3/6 phosphorylation is a consequence of the inhibition of SGT1 function, and the subsequent suppression of NLR-mediated activation of these MPKs. This feedback loop is mentioned in the discussion (lines 437-442).**

8. SGT1 2D phosphorylation enhanced RPS2-triggered cell death in Figure 5A-5C in N. benthamiana. How about the cell death after co-expression SGT1 2D or SGT1 DD with RipAC in N. benthamiana?

**>>Response: Following this point raised by the reviewer, we performed an assay co-expressing RPS2 with the mentioned SGT1 variants and RipAC (see attached the result for the reviewer’s information). Unfortunately, since RipAC is able to suppress the RPS2-triggered cell death in all the conditions (SGT1 WT, 2A or 2D), it is difficult to conclude about this result. We believe that RipAC is most likely affecting the endogenous NbSGT1, which is a common element in all these samples, and therefore partially inhibiting HR in all the conditions. The assay is quite complex, the interpretation would require an excessive speculation, and it is not necessary to reach the conclusions of our study; therefore we prefer to keep it out of the manuscript, but provide it here for reference.**

9. The association of SGT1 2D mutant with RPS2 is reduced in Figure 5D, but the RPS2-mediated cell death is enhanced by SGT1 2D in Figure 5A. As the authors mentioned, the enhanced SGT1 phosphorylation might lead to RPS2 to be released from the complex. Just curious, is there any literature reporting that SGT1 phosphorylation is directly involved in the accumulation, recognition and activation of NLR protein?

**>>Response: The text of the results and discussion section mentions the only studies that report SGT1 phosphorylation to date (references 39-41). In Hoser et al (2013) (ref. 39), the authors show that tobacco SGT1 undergoes phosphorylation in a canonical MAPK target-motif by SIPK, and that phosphor-mimic mutations in this motif alter SGT1 localization (enhanced nuclear localization). However, neither phosphor-dead nor phosphor-mimic mutation could complement the N-mediated resistance to TMV. Similarly, Redkar et al (2015) (ref. 41) found slight alterations in ZmSGT1 localization upon mutation of MAPK-mediated phosphosites. These studies did not allow to conclude the relevance of MAPK-mediated phosphorylation for SGT1 function in NLR-mediated immunity, but the relevant information is cited in different parts of the results and discussion sections (lines 268, 279, 428).**

10. SGT1 phosphorylation is very important for RPS2-mediated cell death and RipAC virulence; and the two phosphorylation sites (S271, T346) located in the SGS region, which is required for interaction with RipAC. So how about the interaction of these two mutants with RipAC compared to the interaction of SGT1 WT with RipAC?

**>>Response: We thank the reviewer for this suggestion. Neither the T346A nor T346D mutation affected the interaction between SGT1 and RipAC. This result is now shown in Figure S10H-I, and mentioned in the text (lines 338-339).**

11. Figure 5B, 5C should include the results of SGT1 AA and DD induced cell death.

**>>Response: We have now included these results in the Supporting Figure S10C.**

12. It seems that knockout of SGT1a or SGT1b in Arabidopsis did not affect Pst DC3000 infection (Azevedo et al, 2006, EMBO journal). How about R. solanacearum?

**>>Response: Upon request by the reviewer, we have performed these infection assays. Surprisingly, although previous studies found no differences in the susceptibility of *Atsgt1* single mutants to *Pto* DC3000 (Holt et al, 2005; Austin et al, 2002), we found that both *Atsgt1a* and *Atsgt1b* mutants were more susceptible to *R. solanacearum* than their respective Ws-0 and La-er wild-type backgrounds, respectively. We have now included this explanation in the text, and the results as new Figures 7A-7D and S12A-B.**

**Part II – Major Issues: Key Experiments Required for Acceptance**
Please use this section to detail the key new experiments or modifications of existing experiments that should be absolutely required to validate study conclusions.

Generally, there should be no more than 3 such required experiments or major modifications for a "Major Revision" recommendation. If more than 3 experiments are necessary to validate the study conclusions, then you are encouraged to recommend "Reject".

Reviewer #1: Major concerns:
1) The authors present that an interaction of AtSGT1s with AtMAPK3 and AtMAPK6，and MAPK-mediated phosphorylation is important for SGT1 function. Do AtMAPK3 and AtMAPK6 directly phosphorylate AtSGT1?

**>>Response: We thank the reviewer for this question. Using our anti-pSGT1 antibody after *in vitro* kinase assays we now show that recombinant AtMAPK3 and AtMAPK6 directly phosphorylate recombinant AtSGT1a and AtSGT1b, and this phosphorylation was stronger in the presence of AtMKK5DD (new Figure 4D). This is now mentioned in the text (lines 291-294).**

2) If AtSGT1 is phosphorylated by AtMAPK3 and AtMAPK6，you could knockout or silence AtMAPK3/AtMAPK6 and NbMAPKs, and test SGT1-mediated immune responses and phosphorylation level in SGT1 in Arabidopsis and N. benthamiana, respectively.

**>>Response: We thank the reviewer for these suggestions. As suggested by both reviewers, we used different complementary approaches to show the association between MPK3/MPK6 and SGT1 phosphorylation. First, we used Arabidopsis DEX:MKK5DD plants; we show that MKK5DD expression, which leads to constitutive activation of MPK3/6, enhances SGT1 phosphorylation (Figure 4D). Moreover, we used Arabidopsis WT plants and mutants with abolished AtMAPK3 and AtMAPK6 activity in *Atmapk6* and *Atmapk3* mutant backgrounds, respectively (Xu et al, Plant J, 2014; Su et al, Plant Cell, 2017), and inoculated it with Pto AvrRpt2. The enhancement of SGT1 phosphorylation observed in WT plants was not observed in the mpk3/mpk6 loss-of-function plants, confirming that SGT1 phosphorylation requires these MAPKs (Figure 4I and 4J). These results are now shown in Figure 4, and the corresponding text has been included in lines 295-308.**

3) The authors show that independent transgenic lines expressing RipAC exhibit enhanced disease symptoms upon soil-drenching inoculation with R. solanacearum. All the data only use WT Col-0 as control, it’s better to find a RipAC mutant or EV as control for the experiments.

**>>Response: All the experiments using RipAC-expressing lines have been performed using homozygous T4 transgenic lines. The fact that they are homozygous is now mentioned in the main text (line 163), and the generation is mentioned in the methods section (line 566). Such lines have been germinated and grown in exactly the same conditions as WT plants, since they do not require antibiotic selection (we have now specified this in the method section, line 566-567). In order to avoid potential insertional effects in infection phenotypes, we used two independent transgenic lines. Since the only difference between these lines and WT plants is the presence of RipAC, we considered that WT are appropriate as control for these experiments.**

4) The authors present that Co-localization of plasma membrane-associated CBL-GFP protein and SGT1-RFP in N. benthamiana (Fig S5C). But it appears that it is difficult to distinguish the precision localization of plasma membrane and the cytoplasm. You could determine their precision localization through wall separation experiments.

**>>Response: We agree with the reviewer that our assays do not show precise localization of SGT1 at the plasma membrane. Although SGT1 co-localizes with the plasma-membrane marker CBL1-GFP (shown by the intensity peaks in Figure S5C), we also observed other red peaks (not overlapping with the green peaks) that correspond to cytoplasmic localization. This correlates with the reported nucleocytoplasmic localization of SGT1, and also to our mention to SGT1 localization in the text (line 182-183). Since we do not claim specific plasma-membrane localization, we believe that the co-localization with an established plasma-membrane marker is sufficient to support the heterogeneous localization of SGT1, in addition to the nucleocytoplasmic localization of SGT1 reported by others and also in this manuscript.**

Reviewer #2: (No Response)

**Part III – Minor Issues: Editorial and Data Presentation Modifications**
Please use this section for editorial suggestions as well as relatively minor modifications of existing data that would enhance clarity.

Reviewer #1: Minor concerns
1) Line 230, change Agrobacterium-mediated transformation to Agrobacterium-mediated transient expression.

**>>Response: Changed. Thanks.**

2) Fig S5F, change cLUC+SGT1b to cLUC-SGT1b.

**>>Response: Changed. Thanks.**

3) It’s better to use Empty vector as control in Fig 5B.

**>>Response: Instead of an empty vector, we considered more appropriate to include a vector that triggers the expression of a heterologous protein, as a more accurate control for Agrobacterium-mediated transient expression.**

4) Line 154, from a 35S constitutive promoter to driven by single or double 35S constitutive promoter.

**>>Response: Explained (single promoter). Thanks.**

5) Fig 3G-J, I don’t know if SGT1-independent NLR protein exists. If it exists, It’s better to use SGT1-independent NLR as negative control.

**>>Response: As far as we know, to date, all NLRs seem to depend on SGT1 for function.**

6) Fig1,in infection experiments, no difference analysis of the data, so the virulence function of RipAC is not accurately proved.

**>>Response: We have reorganized the way we represent the Ralstonia inoculation assays followed by observation of disease symptoms. Main figures (e.g. Figure 1) now show the observation of disease symptoms using composite data from different experiments (the independent data from each replicate is provided in the supporting figures, since these graphs are quite complicated and difficult to visualize, but otherwise useful for reference). Moreover, as usually done in Ralstonia research, we converted the disease index into binary survival data for statistical analysis, which is shown side-by-side with the disease index observation. We now provide the p values resulting from the statistical analysis in the figures, and all the analysis is explained in the figure legends.**

7) Figure 2A,the Western results of Flag IP is not full picture.

**>>Response: The signal in the interaction between RipAC and AtSGT1a was weak and therefore it is shown with longer exposure, together with its own control. We considered more fair and transparent to separate it from the rest of the blot, and we have explained this in the figure legend.**

8) Figure 2B, Split-LUC assay does not have quantification of the luciferase signal.

**>>Response: We considered that, given the result observed in the photograph, this result was qualitative and conclusive, clearly showing the interactions between RipAC and SGT1s. Since we perform the qualitative and quantitative assays differently (either photographing the leaves or measuring luminescence of leaf discs using a microplate reader), we did not perform quantitative measurements of this interaction.**

9) In the process of verifying the interaction between SGT1 and MAPKs, the authors have different results from different methods.

**>>Response: Indeed, different results were obtained in different interaction assays, probably due to the different experimental conditions, and by specific incidences (for example, the very low accumulation of AtMAPK3 in CoIP assays). By targeted CoIP, we found that both AtSGT1a and AtSGT1b associate with AtMAPK4 and AtMAPK6, but not with AtMAPK3 (Figure 4A). In Split-LUC assays, we found direct interaction of both AtSGT1a and AtSGT1b with AtMAPK3 and AtMAPK4, but not with AtMAPK6 (Figures 4B, 4C, and S9). We concluded that AtMAPK4 interacts with AtSGT1a and AtSGT1b, which is reproducible in all the assays. While the results also suggest an interaction of AtSGT1s with AtMAPK3 and AtMAPK6, we cannot conclude that directly given the different results obtained. We explain this in detail in the manuscript text (lines 283-286).**

Reviewer #2: (No Response)
